# Personal News Items

**Published:** 1915-07

**Authors:** 


### Personal News Items.

Dr. M. M. Carrick, of Dallas, is spending some time in New York.

Dr. T. O. Staples and son, of Wylie, will visit the Panama-Pacific Exposition.

Dr. C. F. Smith, of Hemphill, has been appointed county health officer of Sabine county.

Dr. J. L. Burgess, of Waco, has been called to Tennessee by the death of his mother.

Dr. Buchanan late, of San Angelo, now of Big Springs, has opened a branch office at Midland.

Dr. and Mrs. J. H. Stewart, of Stephenville, Texas, are visiting relatives in Silver City, New Mexico.

Dr. W. F. Alexander, of Terrell, has recently been appointed county health officer of Kaufman county.

Dr. Hancock, of Ranger, has been recently called to California, because of the serious illness of his mother.

Dr. E. S. Cox, Galveston County Health Officer, attended the Health Officers' School held in Austin June 14th.

Dr. W. M. Copeland, of Lorraine, has returned from Chicago, where he has been doing post-graduate work in surgery.

Dr. W. H. Pope, Jr., of Trinity, Texas, is rapidly recovering from an operation at the Norsworthy Sanitarium, Houston.

Dr. H. K. Weems has returned to his home in Graham, after spending some time in Chicago taking a post-graduate course.

Dr. and Mrs. Biggs, of West, leave for California in a few days, where they will attend the fair and other points of interest.

Dr. and Mrs. H. E. Stevenson and family, of El Paso, will leave soon for an extended trip to California, Grand Canyon, and the West.

Dr. W. H. O'Banion and Dr. T. B. Coopwood left last week for New Orleans, where they will take some special work in Tulane University.

Dr. and Mrs. S. T. Humphreys, of Nacona, will be away for six weeks. The doctor will do some post-graduate work in the University of Chicago.

Dr. and Mrs. Neathery have returned to their home, in Sherman, from New York, where the doctor has been doing some special work in surgery.

Dr. I. L. McGlasson, of Waco, has been invited to prepare a paper on "Urology," to be read before the Pan-American Medical Congress at San Francisco, June 17-19.

The new hospital building at the Home for Aged Masons, near Arlington, Texas, is nearing completion, and will be opened for the reception of patients about June 15th.

At the annual commencement of the Johns Hopkins University, McDugald K. McLean, of Wolfe City, Texas, was honored with the degree of Doctor of Medicine. He will likely remain as a member of the house staff.

Dr. U. P. Hackney of the asylum staff of physicians at Terrell has resigned and gone to Rochester, Minn., where he will do some special work. Upon his return he will locate with St. Paul's Sanitarium in Dallas, where he will have charge of the X-ray work of the institution.

Drs. Martin E. Taber and M. P. Stone have been appointed as Dallas representatives on the medical staff of the Home for Aged Masons and Their Wives at Arlington, Texas. Drs. Frank Boyd and W. A. Durringer are Fort Worth's representatives on the medical staff.

Dr. Oscar Dowling, of New Orleans, President of the Southern Medical Association, which meets in Dallas this year, has extended invitations to the following physicians of note: General Wm. C. Gorgas, Surgeon General of the United State Army; General Wm. C. Baisted, Surgeon General of the Navy; Dr. Rupert Blue, Surgeon General of the Public Health Service, and Dr. Carey T. Grayson, President Wilson's physician.

---

After eigth months of hard work the New York Medical Society and the State Board of Commerce found fifty-four quacks and arrested them and their assistants, and destroyed their "museums" and "institutes."

---

Miss Katherine Besse, of Des Moines, eighteen years old, ate her first meal after Dr. Francis W. Kirsch had performed an operation upon her. The channel of her throat, the esophagus, was in position, but was entirely useless. Dr. Kirsch began by probing with a slender, pliable steel rod, and soon had the closed places in the esophagus opened and broken apart. As they were broken they were left attached to the new tissue that had gradually grown around the unused tube.

